# Start4All: protocol for evaluating TB screening and diagnostic tests and test algorithms in the community and lower care levels in seven high-burden countries

**DOI:** 10.5588/ijtldopen.26.0080

**Published:** 2026-08-11

**Authors:** R.L Byrne, M.Y.R. Henrion, E.R Adams, S. Banu, J. Bimba, A. Choko, A.J. Codlin, A.I. Cubas Atienzar, T. Garg, V. Iem, S. John, P. MacPherson, M. Sander, V. Santos, S.B. Squire, L.N. Quang Vo, S. Wandiga, J. Creswell, T. Wingfield, on behalf of Start4All Investigators, N. Kontogianni, A. McCoy, E. Worrall, O.M. Akinwande, J. Read, A. Choko, C.K. Banda, C. Mbuli, S.M.M. Rahman, S. Ahmed, A.K. Saha, S. Kabir, M.K.M. Uddin, S. Choudhury, T. Rahman, S. Miah, N.N. Ruhee, K.I.A. Chowdhury, R.Q. Gurgel, S.A. Abdulkarim, M.O. Iwakun, J.A. Agaya, A.O. Okumu, I.A. Omondi, N.T.T. Nguyen, H.T. Nguyen

**Affiliations:** 1 Centre for TB, Liverpool School of Tropical Medicine (LSTM), Liverpool, UK;; 2 Department of Clinical Sciences, Liverpool School of Tropical Medicine, Liverpool, UK;; 3 Malawi Liverpool Wellcome Research Programme, Blantyre, Malawi;; 4 iccdr,b, Dhaka, Bangladesh;; 5 Zankli Research Centre, Bingham University, Karu Nasarawa, Nigeria;; 6 Friends for International TB Relief (FIT), Ha Noi, Viet Nam;; 7 Karolinska Institutet, Department of Global Public Health, Solna, Sweden;; 8 Stop TB Partnership, Geneva, Switzerland;; 9 Janna Health Foundation, Jimeta Yola, Adamawa, Nigeria;; 10 School of Health & Wellbeing, University of Glasgow, Glasgow, UK;; 11 Center for Health Promotion and Research (CHPR), Bamenda, Cameroon;; 12 Fundação de Apoio à Pesquisa e Extensão de Sergipe, São Cristó vão, Brazil;; 13 Kenya Medical Research Institute (KEMRI), Nairobi, Kenya;; 14 Department of International Health, Liverpool School of Tropical Medicine, Liverpool, UK.

**Keywords:** tuberculosis, ACF, CAD, Bangladesh, Brazil, Cameroon, Kenya, Malawi, Nigeria, Viet Nam, facility-based case finding, community-based case finding, vulnerable populations

## Abstract

**BACKGROUND:**

Despite advances in TB care, approximately 2.4 million people with TB remain undiagnosed or unreported each year. A key contributor to these ‘missed millions’ is that major diagnostic gaps persist especially among underserved populations. Developing diagnostic algorithms that are accessible and integrated into health care systems where they are intended to be used is essential to meet WHO End TB targets.

**METHODS:**

Start4All a multi-country, cross-sectional diagnostic accuracy study (in Bangladesh, Brazil, Cameroon, Kenya, Malawi, Nigeria, and Viet Nam) aims to assess and model algorithms using combinations of WHO-endorsed, and experimental TB screening and diagnostic tests across the lowest levels of care and the community, compared to a sputum culture reference standard. Evaluated tools include C-reactive protein tests, computer-aided detection–interpreted chest X-rays, pooled Xpert® MTB/RIF Ultra (Xpert Ultra) testing, and a third generation lipoarabinomannan (LF-LAM) assay.

**DISCUSSION:**

Start4All will generate high-quality evidence to guide policy decisions on the adoption and the integration of non-sputum and laboratory independent tools to accelerate early TB detection and close the treatment gap.

**CONCLUSION:**

A feature of Start4All is its implementation within national TB programmes across seven high-burden countries to ensure that findings are directly translatable to policy and operational decision making. Harmonised protocols, standardised microbiological reference testing, and unified data management systems will enable cross-country comparison and strengthen the external validity of results.

Globally, TB remains the leading cause of death from a single infectious disease, despite being both preventable and curable when detected early. It is estimated that 10.7 million people fell ill with TB in 2024, with approximately 2.4 million of these people never being diagnosed, treated, or reported to national TB programmes (NTPs). This underserved group, often termed ‘the missed millions’, are considered a key driver of the 1.25 million deaths attributed to TB annually.^1,2^ TB affects all countries and age groups, but disproportionately impacts low- and middle-income countries (LMICs); all 30 of the highest TB-burden countries are classified as LMICs and together they account for 90% of the global TB burden. Efforts to end TB have been slow to meet elimination targets and were further impeded by the COVID-19 pandemic.^3^

Significant gaps remain in TB diagnosis, particularly at the lowest levels of the health system, where the majority of people first seek care.^4–6^ In many high-burden countries, reliance on symptom-based screening and poor access to diagnostic tests results in missed or delayed diagnoses. This is particularly true in people living with HIV (PLHIV) and children (people 14 years and younger), who often present with non-specific symptoms and paucibacillary disease,^7,8^ as well as other groups who face barriers in accessing health care, including economic and social.^9^ Diagnostic pathways frequently depend on sputum-based tests that are unavailable or inaccessible in peripheral facilities.^5^ Strengthening diagnostic strategies that combine simplified tools, decentralised technologies, and clear referral pathways at the primary care level is essential to address the TB treatment gap. Combining tests sequentially allows the inclusion of assays that individually might not have sufficient performance but as part of an algorithm capitalises on their strengths whilst mitigating individual limitations. This is especially useful for decentralised TB testing to allow tests that can capture a diverse range of TB symptoms and clinical presentations.

## Relevance of integrated, non-sputum and digital innovations at lower care levels

At lower levels of care, it is essential that technologies can be and are decentralised. Rapid molecular platforms, portable digital radiography systems combined with computer-aided detection (CAD) software for chest X-ray (CXR) interpretation, and biomarker-based assays that can be deployed outside of laboratories all exist, but require an evidence-based approach to maximise their impact and sustainability that is both context and population specific. Lessons should be learnt from the introduction of GeneXpert, which was positioned to revolutionise the way we test TB. However, programmatic evaluations have shown that it has frequently failed to reach peripheral facilities because of cost, infrastructure, and operational barriers, and has not been shown to improve TB treatment outcomes.^10,11^ Investment at the lowest levels of the health system and within the community is not only a matter of health care equity; earlier and more accurate diagnosis prevents disease progression, reduces the risk of severe complications, and can also be cost-saving.^12^ These cost savings are not only within health care systems, but are also important for people affected by TB, by reducing long-term treatment and associated costs.^13^

## Study description and objectives

The Start4All consortium is a Unitaid-funded research project comprising two distinct study phases (https://clinicaltrials.gov/study/NCT05845112). Phase 1 is a prospective diagnostic accuracy study designed to evaluate and model the algorithmic performance of TB screening and diagnostic tests across a diverse range of high-TB-burden countries, settings (e.g., primary health care facilities, district hospitals, and the community), and target populations (e.g., internally displaced people, nomadic groups, people living in informal settlements, those living rurally, urban low-income communities, and children [aged 1–14 years]). The screening and diagnostic tests to be evaluated as part of Phase 1 are pooled testing of sputum by Xpert Ultra, rapid and quantitative C-reactive protein (CRP), CAD-interpreted CXRs, and a third generation LF-LAM (see Supplementary Material 1) in people with presumptive pulmonary TB. Phase 2 aims to implement and evaluate the best performing modelled test algorithms across a subset of countries based on diagnostic performance, cost effectiveness, feasibility, acceptability and scalability. This protocol manuscript will only describe Start4All’s Phase 1 activities.

The objective of Phase 1 of Start4All is to evaluate, by country and setting, the performance of selected tests and model the performance of test algorithms for the diagnosis of TB at the lowest level of health care and within the community in countries with a high TB burden in the general population (Bangladesh, Brazil, Kenya, Nigeria), countries with a high burden of HIV-associated TB (Bangladesh, Cameroon, Kenya, Malawi, Nigeria), and countries with a high burden of drug-resistant TB (Bangladesh, Nigeria, and Viet Nam). A secondary objective will be to select the test algorithms per setting that have the best performance in comparison to the reference standard. Another secondary objective is to evaluate the performance of selected tests and pre-specified algorithms in aggregate analyses, by setting, but across multiple countries for each setting. Participants will be recruited through facility-based case finding (FBCF), including primary health care and district hospitals, and through community-based case finding (CBCF), also referred to as active case finding.

## METHODS

Start4All Phase 1 is a multi-country cross-sectional prospective diagnostic evaluation study of the performance of CRP (rapid diagnostic test and quantitative point of care platforms), CAD-interpreted CXRs, pooled testing of multiple sputum samples with a single Xpert Ultra cartridge, and third generation LF-LAM – individually and as pre-specified diagnostic algorithms. Where a test is not currently part of routine care, the results will not be shared with the clinical team and thus not interfere with routine clinical management.

### Study participants

Adults will be recruited from lowest level health care facilities (primary health care and district hospitals) across all countries and CBCF in Bangladesh, Cameroon, Nigeria, and Viet Nam. Adults are categorised as 15 years of age and above in Bangladesh, Cameroon, Kenya, Nigeria, and Viet Nam and 18 years of age and above in Brazil and Malawi. Children will be recruited from health care facilities (primary health care and district hospitals) from Cameroon and Viet Nam. Children are categorised as 1–14 years inclusive.

### Eligibility and inclusion criteria

Start4All will consecutively recruit adults (all countries) and children (Cameroon and Viet Nam) with presumptive pulmonary TB as characterised by the WHO four symptom screen^14^ (W4SS; cough, fever, night sweats, weight loss) from health care facilities (see Supplementary Material 2 for full symptom criteria). Individuals screening positive and eligible for inclusion will be invited to a private area of the health care facility and provided further details of the Start4All study and, if willing to participate, written informed consent will be sought (Supplementary Material 3). For children and young adolescents, written informed consent will be obtained from a parent or guardian at the time of enrolment and assent signed for those aged 15 years to age of majority in the respective country.

### Community-based case finding

In Bangladesh, Cameroon, Nigeria, and Viet Nam, adults will be consecutively recruited through CBCF initiatives. Based on local context, CBCF will be conducted in informal urban settlements in Bangladesh, rural communities in Cameroon, and amongst nomadic and internally displaced populations in Nigeria and urban low-income communities in Viet Nam. The CBCF will be organised through lung health camps. There will be community sensitisation drives before the health camps to invite people to participate in the camp. All individuals who are interested in being evaluated for lung disease attending the camp will be screened upon contact by both W4SS, under the same parameters as in health care facilities, and by CXR. A posterior-anterior (PA) X-ray will be taken from all individuals and interpreted by CAD software (qXR, Qure.ai, Mumbai, India). Any individuals that have a CAD software abnormality score of ≥0.3, living with HIV and/or a household contact will be eligible for study entry irrespective of symptoms. The threshold of ≥0.3 has been selected based on previous work conducted within our study countries for CBCF activities.^15^

### Exclusion criteria

Individuals will be excluded from the Start4All study during both FBCF and CBCF if they cannot provide written informed consent or an agreement to follow up, are undergoing current anti-TB treatment (counting from the third dose), have received any anti-TB treatment or TB preventative therapy within 6 months prior to enrolment (3 months for children), or are exhibiting any clinical ‘danger signs’. Danger signs include but are not limited to respiratory rate >30/min, and/or fever >39°C and/or pulse rate >120/min and/or unable to walk and/or miliary TB found on CXR (i.e., high clinical suspicion of mycobacteraemia and high likelihood of clinical deterioration).

### Participant flow

For individuals recruited via community-based screening, a CXR will be taken prior to study enrolment, supported by the national NTP as routine practice in CBCF activities. For those recruited via facility-based screening, CXR will occur after study enrolment. Individuals who meet the eligibility criteria will then be invited to a private room within the health care facility, provided with a patient information leaflet, and given an opportunity to ask any questions about participation. They will be ensured their decision to agree or decline the invitation will not negatively impact their routine care. Following written informed consent, individuals will be interviewed by the Start4All study team and a comprehensive case report form (CRF) will be taken (Supplementary Material 3). This will include key demographics, TB history – both personal and household – and data on associated TB risk factors. Participants will then be asked to provide two spot sputum samples 30 minutes apart, whole blood, a CXR (for those enrolled via FBCF), and urine. The Figure outlines the specimen collection procedure and testing conducted as part of the study. For children, where sufficient sputum samples cannot be obtained, per national guidelines, one or more of the following specimens will be taken to allow reference standard testing: induced sputum, gastric aspirate, nasopharyngeal aspirate, and stool. In Cameroon, venous whole blood will be used as this is routine clinical practice, but for all other countries capillary whole blood will be used from a single finger prick sample. For all adults, only one PA CXR will be taken. Thus, for individuals recruited through CBCF, the CXR taken during pre-consent eligibility screening will be used for CAD software processing, while those recruited through FBCF, will have their CXR taken and processed with the CAD software following study enrolment. For children aged <10, an additional lateral CXR image will be taken.

**Figure.**
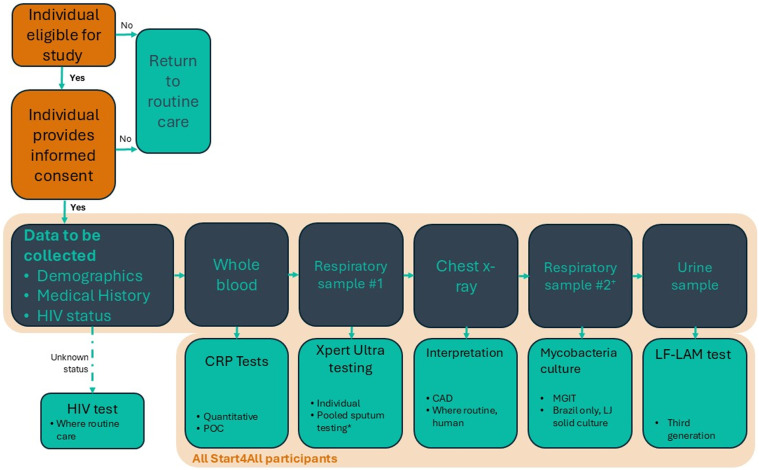
Outline of specimen collection. CRP = C-reactive protein; POC = point of care; CAD = computer-aided detection; MGIT = mycobacteria growth indicator tube.

## TEST METHODS

### Microbiological reference standard (MRS)

The microbiological reference standard in this evaluation will be mycobacteria growth indicator tube culture, for all countries except Brazil, where, as is routine practice, solid culture using Löwenstein–Jensen media will be used. The results of the MRS will not be disclosed to the laboratory personnel performing the experimental test evaluations.

### Routine TB testing

All participants from all countries will have a single sputum Xpert Ultra test performed, with results made available to the clinical team for case management. For children, in addition to culture and Xpert Ultra testing, Cameroon and Viet Nam routinely perform a tuberculin skin test (TST); a Arkay PPD (Japan) and BB-NCIPD (Bulgaria) TB skin test (TST), respectively, results will be recorded but not used as part of reference standard classification. In Bangladesh, Brazil, Cameroon, Kenya, and Malawi, sputum smear microscopy will be performed, at health care facilities as routine practice. The national standard of care and experimental tests introduced as part of this study are outlined in Supplementary Material 4.

### TB case definition

For adults, disease status will be determined through MRS confirmation and will be independent to TB treatment notification, which will not be evaluated in Start4All Phase 1. Children will be classified for the purposes of test evaluation using the ‘updated clinical case definition for classification of intrathoracic tuberculosis in children’.^16^ Where radiological symptoms are to be used for case definition, the corresponding CXRs will be interpreted by a paediatric radiologist from a high-TB-burden country as coordinated by Worldwide Radiology (WWR), an organisation developing radiological capacity in low resource settings. Start4All will not influence the on-site clinical decision making of health care workers related to any participants.

### HIV testing

All individuals will be asked their HIV status on enrolment. A HIV rapid test will be offered to participants in high HIV burden countries (Brazil, Cameroon, Kenya, Malawi, and Nigeria) when their HIV status is unknown or not available in the medical chart, as per national routine practice. In Viet Nam, HIV testing is not routine at any health care centres included as Start4All study sites. In Bangladesh, HIV testing is not routinely performed amongst people with TB nationally. In children <18 months, HIV testing is based on an HIV RNA or DNA nucleic acid amplification test (PCR), following national guidelines. After a positive HIV result, clinical management will follow local guidelines, including counselling and treatment.

### Experimental test selection

As part of Start4All Phase 1, two classes of WHO-recommended TB screening tests will be evaluated: CRP and CXRs interpreted by CAD software. However, both are restricted to use in specific populations, with CRP limited to PLHIV and CXR with CAD in adolescents and adults aged ≥15 years in place of human interpreters. Concurrently, using the molecular WHO-recommended low complexity nucleic acid amplification test (LC-NAAT) Xpert Ultra, the strategy of pooling sputum samples from multiple individuals and testing with one cartridge will also be investigated, hereby known as pooled testing. In Start4All, this will preferentially be a pool ratio of 4:1, but if an odd number of specimens are collected (e.g., not evenly divisible by four) or sufficient specimens are not available, ratios of 3:1 and 2:1 will be performed. Finally, a new (third) generation LF-LAM assay will be evaluated for diagnostic accuracy across the general population and not just in PLHIV, a population which is not currently recommended for LAM testing by WHO. Supplementary Data S4 describes all experimental tests used as part of this study. Experimental test results will not be shared with the clinical team and so will not influence clinical management. The only exceptions are in Cameroon where pooling of sputum is routinely implemented to optimise cartridge use and in Bangladesh where CAD interpretation of CXRs is being routinely used by the NTP.

### Blinding

It is not feasible to blind study staff performing CAD-CXR or CRP, as both tests are conducted in the same room. However, results from CAD-CXR and CRP will not be shared with staff performing Xpert or culture testing, and vice versa. LAM and pooled testing will be carried out by laboratory personnel who are blinded to patient identifiers and, therefore, to all other test results.

## DATA COLLECTION AND ANALYSIS

As a prospective diagnostic accuracy study, reporting will follow the diagnostic accuracy studies (STARD) standard.^17^ The metrics to be included will be sensitivity, specificity, and positive and negative predictive values.

### Sample size justification

TB testing algorithms are tailored by country and target populations; Table 1 gives the number of TB people with TB/number of participants at different prevalence values and different values of precision for 95% confidence interval of point estimate of number of participants with microbiologically confirmed TB identified from each sample population. The sample sizes are chosen to achieve an acceptable level of precision for the estimates of the performance of the both individual TB tests and algorithms. The sample sizes are also suitable for estimating costs and cost-efficiency of the TB test combinations. The calculations in Table 1 have been obtained using the standard Wald normal approximation:

$$n_{unadj}=p\cdot \left(1-p\right)\cdot {\left(\frac{z_{\alpha /2}}{E}\right)}^{2}$$

where *p* = target proportion (sensitivity in our case; *p* = 0.9), E = target precision (or margin-of-error), and z_a/2_ is the (two-sided) critical value from the normal distribution for significance level a (in our case a = 0.05 and z_a/2_ = 1.96). The resulting number n_unadj_ needs to be adjusted for the TB prevalence, p_TB_ (Table 1 shows both n_unadj_ and n_adj_):

$$n_{adj}=\frac{n_{unadj}}{p_{TB}}$$

**Table 1.** Sample size calculations for adults to be recruited in Phase 1 of Start4All (the numbers in each cell are n_unadj_/n_adj_ as explained in the sample size calculation equation).

TB prevalence (%)	Precision					
	5%	6%	7%	8%	10%	15%
	Expected number microbiologically confirmed TB cases/number required in sample					
1	139/13,900	97/9,700	71/7,100	55/5,500	35/3,500	16/1,600
2	139/6,950	97/4,850	71/3,550	55/2,750	35/1750	16/800
3	139/4,633	97/3,234	71/2,367	55/1834	35/1,167	16/533
4	139/3,475	97/2,425	71/1775	55/1,375	35/875	16/400
5	139/2,780	97/1940	71/1,420	55/1,100	35/700	16/320
8	139/1738	97/1,213	71/888	55/688	35/738	16/200
9	139/1,545	97/1,078	71/789	55/612	35/389	16/178
10	139/1,390	97/970	71/710	55/550	35/350	16/160
12	139/1,158	97/809	71/592	55/459	35/292	16/133
15	139/927	97/647	71/473	55/367	35/233	16/107
20	139/695	97/485	71/355	55/275	35/175	16/80
27	139/515	97/360	71/263	55/204	35/130	16/60

Populations with predicted low proportions (<10%) of microbiologically confirmed TB (children, CBCF in Viet Nam) will use the same cross-sectional design, but the number of participants with microbiologically confirmed TB may be purposely enriched by inviting individuals attending adjacent centres to the study centres and/or adding intensified FBCF activities (i.e., recruiting inpatients, as well as ambulatory outpatient). Once included in the study, participants will undergo all TB tests under evaluation in the TB test combination. The number of participants to be recruited per country and target population are shown in the Supplementary Material 6 (SM6), further taking into account different levels of expected attrition (due to loss-to-follow-up, unusable data and other causes):

$$n_{final}=\frac{n_{adj}}{1-p_{attr}}$$

where p_attr_ = expected attrition (expressed as a proportion).

The studies will be analysed individually by countries, and then as a single multi-country evaluation (full details of the model are in Supplementary Material 5). The total sample size to be enrolled varies with the average TB prevalence, target precision, and expected loss-to-follow-up (individuals not completing a diagnostic process) in the individual countries and sites. However, with the assumptions shown in SM6, and assuming an overall proportion of 50% of adult participants being PLHIV, we estimate a sample size of 7,200 PLHIV and 7,200 HIV-negative persons will include at least 548 PLHIV and 548 HIV-negative persons (total 1,096) with a microbiologically confirmed diagnosis of TB. If the recruitment of PLHIV is lower than expected, countries with high burden of HIV-associated TB may enrich recruitment of this population by recruiting in HIV care and treatment clinics and outpatient centres. Similarly, if the TB prevalence amongst study recruits falls below that listed in SM6, countries may adjust case finding activities or focus eligibility criteria to enrich for the recruitment of people with TB.

### Data collected using electronic CRFs

Study data will be collected using electronic case report forms (eCRFs) developed in REDCap,^18,19^ a secure, web-based platform designed and maintained by the Global Health Trials Unit (GHTU). The Databases were designed with built-in range checks, branching logic, and required fields to minimise entry errors and ensure completeness. Paper CRFs were available as an option. Of the eight implementing partners, five will use the REDCap databases designed and hosted by GHTU, two will use REDCap databases built and hosted in-country with the support of data dictionaries supplied by GHTU to ensure uniformity in field and variable naming, and country partners will use a locally developed and locally hosted data collection software,^20^ with data being transferred to GHTU for bulk upload into the central REDCap database. Quantitative data will be captured for all enrolled participants, including demographic characteristics, medical history, clinical examination findings, and results from diagnostic tests. Laboratory data, including microbiological reference standard results, will be entered into separate eCRFs. All digital CXR image files will be linked to anonymised study IDs, processed and stored separately from the study data. Data entry will be performed by trained study nurses, community health workers, and laboratory staff at participating sites across Bangladesh, Brazil, Cameroon, Kenya, Malawi, and Viet Nam. Data will be entered in real time where possible. Local study teams will perform routine checks, and the GHTU team will conduct centralised monitoring and data validation. All records will be linked to unique participant identifiers, with personal identifiers stored separately from study data to preserve confidentiality.

### Sample storage

Where sufficient budget is available, sputum samples with at least 0.5 ml of residual specimen and urine with at least 0.2 ml residual specimen will be eligible for storage for 5 years and, if appropriate, tested locally. Venous blood as serum or plasma may also be stored when sufficient volume remains. All samples will be stored in deep freezers (−80°C) until testing. Methods for sample preservation will follow standard operating procedures developed in consultation with FIND for sample storage. Anonymised CXRs will be stored on the Box server and potentially used for product development and evaluation.

### The establishment of a paediatric training CXR library

Anonymised CXRs from children and young adolescents will be annotated and reviewed by WWR and matched with MRS and TB definition categorisation to establish a library of images that can be used for CAD software development. The library will be made available to software developers, subject to global access terms.

### Study outcomes and endpoints

In this 4-year project, the Start4All team will work towards three portfolio-level outcomes, which focus on: 1) Accelerating the introduction and adoption of existing TB diagnostic tools and novel TB test combination packages at primary health care level and outreach approaches to increase access to timely detection and linkage to care in LMICs; 2) Developing the conditions for sustainable and equitable access to TB diagnostics tools and TB test combination packages integrated within primary health care; and 3) Strengthening global alliances and national partnerships to enable scale-up. This protocol will work towards outcome 1 with the following study endpoints:

### Primary study endpoint

Generation of robust estimates of the diagnostic accuracy and performance of TB tests and TB test combinations in primary health care settings and key vulnerable populations (KVPs).

### Secondary study endpoints

- Estimates of the predicted performance of TB test combinations and how they perform in specific populations.
- Selection of the optimal TB test combinations for implementation and scale-up in primary health care settings and KVPs based on their feasibility, acceptability, and modelled accuracy and cost-efficiency.

### Study vigilance

Study vigilance procedures define adverse events (AEs) as any unfavourable clinical or biological occurrence during study participation, regardless of causality, and serious adverse events (SAEs) according to International Council for Harmonisation criteria (e.g., death, life-threatening events, hospitalisation, disability, congenital anomaly, or other medically important events). Severity will be graded using the Division of AIDS toxicity table,^21^ and investigators will assess causality in relation to study procedures and concomitant treatments. Given the low-risk, non-interventional nature of this diagnostic study, only limited AEs are expected and routine AE reporting will be restricted to events associated with gastric aspiration in children only. SAE reporting will be limited to deaths and life-threatening events, which must be reported promptly to the sponsor and relevant authorities.

### Ethical statement

Ethics approval has been granted for Phase 1 only (WHO ethical review committee approval ERC.0003921). Oversight is provided by country ethical research committees (ERCs), the Liverpool School of Tropical Medicine (LSTM), and Stop TB Partnership, shown in Table 2. The potential risks and benefits to participants are outlined in the patient information leaflet (Supplementary Material 3). Quality assurance is overseen by LSTM with all countries providing source data where possible from all assays.

**Table 2.** Master and country ethical research committees’ identification codes.

Body	Number
LSTM REC	22-084
WHO ERC (master protocol)	ERC.0003921
WHO ERC Bangladesh	ERC.0004062
WHO ERC Brazil	ERC.0004061
WHO ERC Cameroon	ERC.0004014
WHO ERC Kenya	ERC.0004079
WHO ERC Malawi	ERC.0004033
WHO ERC Nigeria	ERC.0004062
WHO ERC Viet Nam	ERC.0004084

LSTM = Liverpool School of Tropical Medicine; WHO = World Health Organization; REC = research ethics committee; ERC = ethical research committee.

## CONCLUSIONS

By assessing combinations of WHO-endorsed and experimental screening tools, Phase 1 of Start4All will provide evidence to inform how these innovations can be integrated into practice to improve diagnostic access and efficiency. Start4All’s focus on algorithmic testing reflects a shift from single-test evaluation toward an integrated diagnostic ecosystem, consistent with WHO’s call for evidence-based test combinations that balance accuracy, affordability, and feasibility across levels of care.^22^ A defining feature of Start4All is its implementation within NTPs across seven high-burden countries. This approach ensures that findings are directly translatable to policy and operational decision making. Harmonised protocols, standardised microbiological reference testing, and unified data management systems will enable cross-country comparison and strengthen the external validity of results. Importantly, by embedding the study in routine service delivery environments, rather than controlled research settings, Start4All captures real-world operational challenges and enablers, providing policy-relevant insights into how diagnostic innovations can be sustainably scaled.

The inclusion of children, PLHIV, and other target populations reflects the TB community’s priority to address inequities in TB detection.^23^ Evaluating both sputum and non-sputum-based tests acknowledges the diagnostic limitations of sputum-dependent algorithms in these populations. The inclusion of CAD interpreted CXRs is particularly relevant for countries seeking to decentralise radiographic screening to lower care levels and community settings where radiologists are scarce and CXR interpretation quality is likely a challenge. These insights will inform global and national guidance on the role of digital health tools, non-sputum diagnostics, and optimised laboratory processing (Xpert Ultra pooling) in expanding equitable access to TB screening and diagnosis. Start4All will also contribute to the global evidence base on diagnostic pathways and cost-effectiveness. The inclusion of acceptability, feasibility, and implementation assessments ensures that recommendations extend beyond technical accuracy to include considerations of user experience, workflow integration, and health system readiness, all critical determinants for policy adoption.

Conducting this evaluation across diverse epidemiological and programmatic contexts presents challenges. Variations in HIV prevalence, health care infrastructure, and workforce capacity may influence diagnostic performance and operational feasibility. Start4All mitigates these limitations through rigorous quality assurance procedures, structured training, and cross-country coordination, ensuring consistency in implementation. While experimental test results are not used for clinical management in Phase 1, this design safeguards methodological integrity and provides the evidence foundation for future work. Start4All supports the broader goal of accelerating progress toward universal access to TB diagnosis and care, and ending TB as a public health threat by 2035. It is anticipated that the outcomes from this study will provide policymakers, implementers, and global health partners with a robust evidence base to guide the rational integration of novel diagnostic technologies into TB screening and diagnostic algorithms.

## Supplementary Material
