# Supplementary material for: Start4All: protocol for evaluating TB screening and diagnostic tests and test algorithms in the community and lower care levels in seven high-burden countries
Source: IJTLD Open. 2026 Aug 11;3(8):526–33. doi: 10.5588/ijtldopen.26.0080 (PMC13460432; doi:10.5588/ijtldopen.26.0080)
Supplement: Supplementary file 1 [file ijtldopen26-0080_supplementarydata1.pdf]

Supplementary material 1: Inclusion and Exclusion Criteria for Start4All

Supplementary material 1: **The inclusion and exclusion criteria based on age, HIV and ART status for the Start4All consortium** recruiting in Bangladesh, Brazil, Cameroon, Kenya, Malawi, Nigeria and Viet Nam.

Adults<br>15 years and above		Young adolescents<br>10 years – 14 years		Children<br>9 years and below	
HIV- or PLHIV on ART	PLHIV not on ART	HIV- or PLHIV on ART	PLHIV not on ART	HIV- or PLHIV on ART	PLHIV not on ART
Written informed consent<br>***AND***<br>*Presumptive TB based on at least 2 W4SS:*<br>*-Fever*<br>*-cough*<br>*-Weight loss*<br>*-Night sweats*<br>***OR***<br>*Presumptive TB based on a cough of 2 weeks or more or at least 1 W4SS:*<br>*-Fever*<br>*-Weight loss*<br>*-Night sweats*<br>***OR***<br>*A cough*	*Written informed consent*<br>***AND***<br>*PLHIV (irrespective of CD4)*<br>***AND***<br>*Presumptive TB based on at least 1 W4SS:*<br>*-Fever*<br>*-cough*<br>*-Weight loss*<br>*-Night sweats*	*Written informed consent from parent/guardian*<br>***AND***<br>*Presumptive TB based on at least 3 W4SS:*<br>*-cough*<br>*-fever*<br>*-night sweats*<br>*-weight loss*<br>***OR***<br>*A cough of 2 weeks or more*<br>***OR***<br>*Fever > 10 days*<br>***OR***<br>*-failure of broad-spectrum antibiotics for treatment of pneumonia to improve symptoms & signs after 10 days*	*Written informed consent from parent/guardian*<br>***AND***<br>*A cough of any duration and at least 1 other W4SS:*<br>*-fever*<br>*-night sweats*<br>*-weight loss*<br>***OR***<br>*All of the following symptoms:*<br>*-Fever*<br>*-Weight loss*<br>*-Night sweats*<br>***OR***<br>*A cough of 2 weeks or more*<br>***OR***<br>*Fever > 10 days*<br>***OR***<br>*-failure of broad-spectrum antibiotics for treatment of pneumonia to*	*Written informed consent from parent/guardian*<br>***AND***<br>*A cough of 2 weeks or more*<br>***OR***<br>*Fever > 10 days*<br>***OR***<br>*Weight loss in the last 3 months (based on z-score)*<br>***OR***<br>*Failure to thrive in the last 3 months (based on z-score)*<br>***OR***<br>*-failure of broad-spectrum antibiotics for treatment of pneumonia to improve symptoms & signs after 10 days*	*Written informed consent from parent/guardian*<br>***AND***<br>*A cough of any duration and at least 1 other W4SS:*<br>*-fever*<br>*-night sweats*<br>*-weight loss*<br>***OR***<br>*Contact with a known TB case and at least 1 other W4SS:*<br>*-fever*<br>*-night sweats*<br>*-weight loss*<br>***OR***<br>*A cough of two weeks or more*<br>***OR***<br>*Fever > 10 days*<br>***OR***<br>*Weight loss in the last 3 months (based on z-score)*

of 2 weeks or more			*improve sympt oms & signs after 10 days*		***OR***<br>*Failure to thrive in the last 3 months (based on z-score)*<br>***OR***<br>*-failure of broad-spectrum antib iotics for treatment of pneumonia to improve sympt oms & signs after 10 days*
Exclusion: Current (counting from 3rd dose) or previous (60 days) TB treatment or TPT in the past 6 months or No consent.		*Exclusion: Current (counting from 3rd dose) or previous (60 days) TB treatment or TPT in the past 3 months or No consent.*			

Legend: ART = antiretroviral therapy; HIV- = a person who is known to be HIV negative or not known to be HIV positive; PLHIV = person living with HIV; TPT = TB preventive therapy; and CD4 = CD4 T Lymphocyte Count; W4SS = World Health Organization Four Symptom Screen consisting of cough of more than two weeks, fever, night sweats and weight loss. Z score is a statistical measurement that quantifies how far a child's anthropometric measurement (such as weight-for-age, height-for-age, or weight-for-height) deviates from the median value of a healthy, WHO-standardized reference population of the same age and sex to indicate a failure to thrive or grow.

START-4ALL Version 1.2 – 2nd February 2023

[YOUR INSTITUTIONAL LETTERHEAD]

Assent and Patient Information Sheet for children and parents participating in the START 4 All study for the diagnosis of tuberculosis

This assent form and parent information sheet are for children attending primary health care and district hospital clinics, and who we are inviting to participate in a study for the diagnosis of tuberculosis called "START 4 ALL".

The Study coordinators are Professor Bertie Squire (UK) and Dr. Emily Adams (UK) from the Liverpool School of Tropical Medicine and Dr. Jacob Creswell (Switzerland) from the Stop TB Partnership. The study coordinators in each of the participating countries are **Dr. John Bimba** (Zankli Research Centre, Nigeria), **Dr. Stephen John** (Janna Research Foundation, Nigeria), **Dr. Melissa Sander** (Centre for Health Promotion, Cameroon), **Dr. Victor Santos** (Federal University of Sergipe, Brazil), **Dr. Andrew Codlin** (Friends for International Tuberculosis Relief, Vietnam), **Dr. Anjan Saha** (icddr,b, Bangladesh) and **Dr. Stephen Wandinga** (Medical Research Institute, Kenya).

The study sponsor is the Liverpool School of Tropical Medicine, Liverpool, UK.
The study funder is UNITAID.
The full title of the study is "Start taking action for TB diagnosis" (called START – 4 All).

This Informed Consent Form has four parts:

- Parent Information Sheet (to share information about the study with parents of the child)
- Certificate of parental consent (for signatures if the child and parent agree to take part)
- Child Information Sheet (to share information about the study with children)

You will be given a copy of the full Parent Informed Consent and the child Assent Form.

PART I: Information Sheet for parents of children being invited to participate

Introduction: We are doing a study to assess new ways of diagnosing tuberculosis (TB). The study includes children and I would like to give you information about the study before we invite you and your child to be part of the study.

Who is doing the study? This is research is being done in six countries in collaboration with a university in the United Kingdom and children are being invited in Cameroon and Vietnam. In Cameroon the study is coordinated by Dr Melissa Sander and in Vietnam the study is coordinated by Andrew Codlin.

Purpose of the study. TB is caused by a germ which most often infects the lungs. To diagnose TB in adults, we usually search for TB germs in sputum using laboratory tests. However, these tests are not perfect, and sometimes the germ is missed by the test and people with TB are given a false negative diagnosis – or in other words, their disease is missed. TB in children is more difficult to diagnose than in adults, because young children swallow and don't spit the phlegm and we often need to search for the germ in stools, urine and stomach fluids. In this study, we are researching ways to improve TB diagnosis in adult and children. We aim to bring new TB tests out of the laboratory so that results are returned faster, use combinations of new tests to get more reliable results and see whether the diagnostics also work in children.

Participant selection. We are inviting all adults and children who have signs or symptoms of TB to participate in the study, unless they are currently on TB treatment or were recently treated for TB. Children attending the Heath Facilities of Mbingo Baptist Hospital, Mboppi Baptist Hospital, Nkwen Baptist Health Centre and Mutengene Baptist hospital in Cameroon and children attending the Pham Ngoc Thach Hospital, Ho Chi Minh City and Hai Phong Lung Hospital in Vietnam will be invited to participate in the study.

Voluntary participation. Your and your child participation is entirely voluntary. If you or your child choose not to participate, this clinic's services and your medical care will not change. You can also withdraw participation at any point during the study, even if you had agreed earlier on.

What do participants have to do? We will ask you a few questions about your child's general health and will measure his/her height and weight. We will then ask him/her to provide several samples, including sputum, urine, and blood from a finger prick, and to take a chest X-ray.

<u>To collect sputum</u>, if your child is old enough to spit sputum, we will ask him/her to breathe deeply and cough up sputum from the lungs. After 30-60 minutes, we will ask for a second sputum sample. These specimens will be tested by smear microscopy, the Xpert MTB/RIF Ultra assay, and culture which can detect TB germs in your body. Young children often cannot spit sputum and then staff need to obtain a sample of swallowed sputum from the stomach. To take samples from the child's stomach fluids requires inserting a tube through the nose [like this], and then fluid is taking out using a syringe [like this]. This procedure can cause some discomfort. The risk is minimal but in some rare cases can cause respiratory distress which will revert within 30-60 minutes. Also, some hospitals can also obtain samples by using an aereosol (like a steam) which breath by the child using a face mask. In our location we would try to obtain sputum by [cough, gastric aspirate, induced sputum, nasopharyngeal swabs

<u>To collect urine</u>, we will ask you to collect a urine sample when you child want to urinate. This sample will be used to do a Fuji LAM test.

To collect blood, we will collect 4-6 drops of blood from a finger prick of by taking the blood from a vein if the clinic has requested the laboratory for other tests. Blood will be used to measure your C-reactive protein (CRP) levels, which can detect inflammation suggestive of TB.
To take a chest X-ray, we will ask to remove all metal objects from your child and to stand in front of an X-ray machine. The chest X-ray will allow us to see if there is damage to the lungs that suggest TB. Most tests will be conducted here at the clinic. However, some of the study's tests are only available at a laboratory in [NAME, location]. We will send the samples there for testing.

The treatment your child would receive. The decision to treat your child for TB will be made by clinic staff based on the sputum test results. We will not return the urine or blood test results because we must first make sure these tests are working properly before they are used to guide TB treatment. The TB treatment offered to study participants will be the same as those not taking part in the study and will follow national guidelines.

Storage of samples and future use. This study will take place over four years. It is possible that new TB tests become available during this time, and we would like to assess these future tests. For this reason, we would like to ask you to donate your child samples, which would allow us to keep any left over urine and sputum you give us. If you agree, we will store any leftover sample in laboratory freezers after completing all the current tests. Samples maybe tested locally or shipped to external collaborators. We would store the samples for a maximum of five years and would only use them if new TB tests are included in the study. If you do not want us to store the samples, please let us know and we will discard any leftover specimens after testing. Your decision on samples storage does not affect your treatment.

Side Effects. If you decide your child can participate in this study, your child will receive a chest X-ray and a give a blood sample. These two procedures can have some unwanted effects.

- X-rays produce a small amount of radiation. However, the benefit of looking into the lungs outweighs the risks of radiation exposure. X-rays may cause some harm during pregnancy and it is important to let us know if you may be pregnant before taking the chest X-ray to make sure you are no exposed while taking your child's X-Rays.
- Blood will be collected using a finger prick or from a vein using a syringe. Unwanted effects include local pain and the possibility of an infection. While the likelihood of an infection is very low, you should still be aware of the possibility. We will prevent infections by cleaning the area before drawing blood. We will provide appropriate treatment and provide care as needed if you develop an infection.

Benefits. If you decide your child can participate in this study, your child will receive the following benefits: We will test the sputum sample with culture, which is currently the most accurate test for finding TB germs, and it is not routinely used for TB detect because of its costs and the time it takes (up to two months) to get the results. If you are diagnosed with TB, we will facilitate linkage to appropriate TB care. Your participation in the study will help us answer questions about the best way to diagnose TB and future generations will benefit of the knowledge generated by this study.

Reimbursements. We will provide a standard transport stipend of [local agreed stipend]. All of the TB tests which are part of this study will be provided free-of-cost.

Confidentiality. All information you provide to the study will be considered confidential. It will be stored in a secure computer database and only study staff will have access to the data. Only anonymized data will be shared with the study team from the partner countries. All information will

be labelled with a unique number instead of your and your child's name. The master list linking participant names and study numbers will be kept under lock and key and only accessed by your country lead study investigators.

Sharing the Results. Once we finish the study, we will share study findings with local stakeholders. Then study findings will be disseminated more publicly, at conferences and in scientific publications. Your confidential information will never be shared.

Who to Contact. If you have any questions you may ask them now or later, even after the study has started. If you wish to ask questions later, you may contact any of the clinic staff, or call the following person:

- In Cameroon: Dr Melissa Sander, +237 94721484; email: melissa.sander@gmail.com
- In Vietnam: Mr Andrew Codlin, +84 1652512847, email: andrew.codlin@tbhelp.org
-

This proposal has been reviewed and approved by multiple research ethics committees including the National Ethics Committee on Human Health Research in Cameroon and the Pham Ngoc Thach Hospital, Ho Chi Minh City and Hai Phong Lung Hospital in Vietnam; the United Kingdom and Switzerland to make sure that study participants are protected from harm. If you wish to find out more about the study's ethical approvals, contact the name listed above.

You can ask me any more questions about any part of the research study, if you wish to. Do you have any questions?

PART II: Certificate of Consent

I have read the information in Part 1, or it has been read to me. I have had the opportunity to ask questions about the study and any questions that I asked have been answered to my satisfaction. I voluntarily consent my child to participate in this study.

Print name of parent ____________________

Signature of parent ____________________

Date ____________________ Day/month/year

If the parent is illiterate

A literate witness must sign (if possible, this person should be selected by the parent and should have no connection to the study team). Parents who are illiterate should include their thumb-print.

I witnessed the accurate reading of the consent form to the parent of the potential participant, and the parent has had the opportunity to ask questions. I confirm that the individual has freely given consent.

Print name of witness ______________________ AND Thumb print of participant

Signature of witness ______________________

Date ______________________ Day/month/year

Statement by the person taking consent

I have accurately read out the information sheet to the parent of the potential participant, and to the best of my ability, made sure that the parent understands that the following will be done:

1. Provide sputum, blood and urine samples for TB testing
2. Take a chest X-ray
3. Keep left over samples for future testing for TB and other germs

☐ I agree that the samples and data generated from my child's participation in this study may be used for additional scientific studies on TB or other germs.

I confirm that the parent has been given an opportunity to ask questions about the study, and all the questions asked by the parent have been answered to the best of my ability. I confirm that the individual has not been coerced into giving consent, and the consent was freely and voluntarily given. A copy of this Informed Consent Form has been given to the parent .

Print name of person taking the consent_________________________

Signature of person taking the consent _________________________

Date _________________________ Day/month/year

PART III. INFORMED CONSENT/ASSENT FORM

Participant information sheet for minors aged ≥ 15

- **Introduction**: We are doing a study to find better ways to diagnose tuberculosis (TB). We would like to give you information about the study and invite you to consider taking part.
- **Who is doing the study?** The study is coordinated by Dr Melissa Sander in Cameroon and Andrew Codlin in in Vietnam in collaboration with a university in the UK.
- **What is TB and why are we doing this study.** TB is caused by a germ that infects the lungs. To find out if a person has TB, we look for the germ in the lab. The lab tests are not perfect and sometimes miss the germ and this is why we are trying to improve how to use the tests.
- **Participant selection**. Children attending Mbingo, Mutengene and Mboppi Baptist Hospitals or Nkwen Baptist Health Centre in Cameroon and children attending Pham Ngoc Thach Hospital, Ho Chi Minh City and Hai Phong Lung Hospital in Vietnam who may have TB are being invited.
- **Voluntary participation.** You are free to decide whether to participate or not. If you choose not to participate, this services and medical care will not change. You can change your mind and stop participating at any time during the study, even if you had agreed earlier on.
- **What do participants have to do?**We will ask you or your parent a few questions about your health and will measure your height and weight. We will ask for samples of sputum, urine, and blood and take a chest X-ray. Sample collection will take up to 90 minutes of your time.
- <u>To collect sputum</u>, if you can spit sputum, we will ask you to collect sputum in a plastic cup. After 30-60 minutes, we will ask for a second sample. The lab will look for the germ using the microscope and other tests called Ultra, and culture. Children often cannot spit phlegm and we may search for the germ in stools, urine and stomach fluid.
- <u>To collect urine</u>, we will ask you to collect urine in a plastic container.
- <u>To collect blood</u>, we will collect 4-6 drops from a finger prick or a vein.
- <u>To take a chest X-ray</u>, we will ask you to stand in front of the X-ray machine.
- <u>You may be invited to come up to provide more sample if we are unable to collect all in the first visit.</u>
- **The treatment you would receive**. The clinic staff will decide whether to treat you for TB or not. The treatment offered to you will be the same as those not taking part in the study. **Storage of samples and future use**. We will not store samples from children.
- **Side Effects.** If you decide to participate, we will take a chest X-ray and blood. X-rays produce a small amount of radiation, but we feel the benefit of looking into the lungs outweighs the risk of radiation. Blood will be collected using a finger prick or a vein. This can be slightly painful and could result in an infection. These risks however are very low and we will make sure your skin is clean to avoid infections. We would provide treatment and care if you develop an infection.
- **Benefits**. If participate we will test the sputum with some tests (like culture), which are not routinely used because they are expensive and takes long (two months) to get the results. If have TB, we will take you to the clinic where you can have TB treatment. Your participation will help us to find out the best ways to diagnose TB, which will help other people in the future.
- **Reimbursements**. We will provide you with a transport stipend. All TB tests will be free-of-cost.
- **Confidentiality**. All information will be stored in a computer. Only study staff will have access to the data. No personal information will be shared with other people. We will share the

findings at conferences and in scientific publications, but confidential information will not be shared.

- **Who to Contact**. If you have any questions you can ask them now later. If you wish to ask questions later, you can contact the clinic staff, or call:

- In Cameroon: Dr Melissa Sander, +237 94721484; email: melissa.sander@gmail.com
- In Vietnam: Mr Andrew Codlin, +84 1652512847, email: andrew.codlin@tbhelp.org

For complaints, please email S4A@lstmed.ac.uk

This proposal has been approved by research ethics committees, including the National Ethics Committee on Human Health Research (Cameroon) and the Pham Ngoc Thach Hospital, Ho Chi Minh City and Hai Phong Lung Hospital (Vietnam) to make sure that all participants are protected from harm.

PART IV. INFORMED CONSENT/ASSENT FORM

Participant information sheet for minors aged < 15

- **Introduction**: We are doing a study to find better ways to diagnose a bug called tuberculosis (TB). We would like to give you information about the study and invite you to consider taking part.
- **Who is doing the study?** The study is coordinated by Dr Melissa Sander in Cameroon and Andrew Codlin in in Vietnam in collaboration with a university in the UK.
- **What is TB and why are we doing this study.** TB is caused by a germ that infects the lungs. It can make you ill. The lab tests are not perfect and sometimes miss the germ and this is why we are trying to improve how to use the tests.
- **Participant selection**. Children attending Mbingo, Mutengene and Mboppi Baptist Hospitals or Nkwen Baptist Health Centre in Cameroon and children attending Pham Ngoc Thach Hospital, Ho Chi Minh City and Hai Phong Lung Hospital in Vietnam who may have this bug are being invited.
- **Voluntary participation.** You are free to decide whether to participate or not. If you choose not to participate, your medical care will not change. You can change your mind and stop participating at any time during the study, even if you had agreed earlier on.
- **What do participants have to do?** We will ask you or your parent a few questions about your health and will measure your height and weight. We will ask for samples of sputum, urine, and blood and take photo of your lungs called (X-Ray). Sample collection will take up to 90 minutes of your time.
- <u>To collect sputum</u>, if you can spit sputum, we will ask you to collect sputum in a plastic cup. After 30-60 minutes, we will ask for a second sample. The lab will look for the germ using the microscope and other tests to find the bug.
- <u>To collect urine</u>, we will ask you to collect urine in a plastic container.
- <u>To collect blood</u>, we will collect 4-6 drops from a finger prick or a vein.
- <u>To take a chest X-ray</u>, we will ask you to stand in front of the X-ray machine.
- <u>You may be invited to come up to provide more sample if we are unable to collect all in the first visit.</u>
- **The treatment you would receive**. The clinic staff will decide whether to treat you for TB or not. The treatment offered to you will be the same as those not taking part in the study.
- **Storage of samples and future use**. We will not store samples your samples.
- **Side Effects.** If you decide to participate, we will take a chest X-ray and blood. X-rays produce a small amount of radiation, but we feel the benefit of looking into the lungs outweighs the risk of radiation. Blood will be collected using a finger prick or a vein. This can be slightly painful and could result in an infection. These risks however are very low and we will make sure your skin is clean to avoid infections. We would provide treatment and care if you develop an infection.
- **Benefits**. If participate we will test your samples to find the bug, which are not routinely used because they are expensive and takes long (two months) to get the results. If you have the bug, we will take you to the clinic where you can have TB treatment. Your participation will help us to find out the best ways to diagnose TB, which will help other people in the future.
- **Reimbursements**. We will provide you with a transport stipend. All TB tests will be free-of-cost.
- **Confidentiality**. All information will be stored in a computer. Only study staff will have access to the data. No personal information will be shared with other people. We will share the

findings at conferences and in scientific publications, but confidential information will not be shared.

- **Who to Contact**. If you have any questions you can ask them now later. If you wish to ask questions later, you can contact the clinic staff, or call:

- In Cameroon: Dr Melissa Sander, +237 94721484; email: melissa.sander@gmail.com
- In Vietnam: Mr Andrew Codlin, +84 1652512847, email: andrew.codlin@tbhelp.org

For complaints, please email S4A@lstmed.ac.uk

This proposal has been approved by research ethics committees, including the National Ethics Committee on Human Health Research (Cameroon) and the Pham Ngoc Thach Hospital, Ho Chi Minh City and Hai Phong Lung Hospital (Vietnam) to make sure that all participants are protected from harm.

PART V. INFORMED ASSENT FORM FOR CHILDREN

Project title: Start 4 All

Child (or if unable, parent on their behalf)/young person to circle all they agree with:

1. Do you understand what this project is about? Yes/No
2. Have you asked all the questions you want? Yes/No
3. Have you had your questions answered in a way you understand? Yes/No
4. Are you happy to take part? Yes/No

If any answers are 'no' or you don't want to take part, don't sign your name! If you do want to take part, you can write your name below

I confirm I have been informed about the study and received a copy of the information sheet and the Assent Form. I understood the information. I have been given sufficient information concerning the conditions, the duration of the study and any possible inconvenience to me. I have received sufficient time to consider the information and to ask questions, to which I have received satisfying answers by (**name of person explaining the study**). I agree that the information and samples may be used for additional scientific studies on TB or other germs. I may choose to leave the study at any time and will not be penalized or prejudiced in any way.

☐ I agree that my samples may be stored after completion of this study.

Date: ______________ Name:

____

Signature (or thumbprint) of child:

____

Name of the witness:

____

Signature:

____

Name of parent/legal representative

____

Signature (or thumbprint) of parent

____

Part destined to the investigator's team

I, the undersigned, ……………………………………………………………. confirm that I have informed the participant (and/or the legal representative) about all the relevant aspects of this study. I confirm that he/she has consented voluntarily to participate in the study.

Date: ____

Signature: ____

C-reactive protein (CRP)

CRP is a blood inflammatory marker that is elevated in the presence of an infection, inflammatory or neoplastic (cancer) conditions, including TB. CRP as a screening tool is recommended by the WHO for PLHIV at a threshold of 5mg/L.

CRP Semi-quantitative lateral flow assay

The lateral flow test used in this study was Right Sign CRP Semi-Quantitative Rapid Test (Hangzhou Biotest Biotech Co., Ltd. Hangzhou, China). Clean the finger of the patient with an alcohol pad. Let it dry and use a lancet for the fingerstick. Fill the capillary tube and transfer approximately 10µl of fingerstick whole blood specimen to the dilution tube with buffer. Invert the tube several times to mix the specimens well. Add 3 drops of diluted specimen to the specimen well. Wait for the coloured line(s) to appear. The test result should be read at 5 minutes (do not interpret the result after 8 minutes).

Interpretation: Positive result: One coloured line appears in the control region (C). One or more coloured lines appear in the test region (T), see table 3. Negative result: One colour line appears in the control region (C). No apparent purple line appears in the test region (T).

Table 1 The interpretation of Right Sign CRP Semi-Quantitative Rapid Test

**Test line (T)**	**Semi quantitative result**
No line	CRP level is lower than 10mg/L
Only T1 appears	CRP level is 10mg/L at least
Only T1 and T2 appear	CRP level is 40mg/L at least
T1, T2 and T3 appear	CRP level is above 80 mg/L

CRP quantitative POC devices

CRP quantitative POC devices are platforms for quantitative determination (in mg/L) of CRP circulating in blood with a time to result of 5 minutes. The CRP POC devices used in Start4All were selected based on local registration and procurement and whether these were already in used in the country partners. In Bangladesh, Brazil, Cameroon and Viet Nam, the quantitative POC device used was ichroma™ CRP (Boditech Med Inc.'s Technical Services, Gang-won-do, Korea); in Kenya and Malawi, the quantitative POC used was the Getein 1160 Immunofluorescence Quantitative Analyzer (Getein Biotech, Inc., Nanjing, China) and in Nigeria was the CUBE-S photometer (Eurolyser Diagnostica GmbH, Salzburg, Austria).

The ichroma™ CRP is a fluorescence-based assay where 10 µl of sample (blood, serum or plasma) are incubated with the detection buffer, incubated for 3 minutes at room temperature and loaded into the cartridge. Cartridge is loaded into the ichroma™ CRP fluorescence reader, and the CRP concentration is automatically displayed in the screen.

The Getein 1160 Immunofluorescence Quantitative Analyzer is a fluorescence-based analyser were 10 µl of sample (blood, serum or plasma) are mixed with the sample diluent, mixed gently and thoroughly. Then 100 µl of the sample mixture is transferred into the sample port of the test card of the hs-CRP+CRP Fast Test Kit, on which is then inserted into the Getein 1160

analyzer. The instrument will count down the reaction time (3 minutes) and automatically test the card after the reaction time has elapsed.

The CUBE-S photometer measures at 546 nm (serum) or 700 nm (blood and serum) the antigen-antibody reaction between antibodies to human CRP bound to polystyrene particles. Briefly, 5µl of sample are loaded into a cuvette that is inserted into the CUBE-S photometer to measure, and the CRP concentration is automatically displayed in the screen.

Urinary lipoarabinomannan (LAM)

LAM is a major structural carbohydrate antigen of the outer surface of *Mycobacterium tuberculosis (M.tb)*, the causative agent of tuberculosis disease. LAM testing is a rapid, point of care diagnostic that is able to detect secreted LAM in urine. Currently, LAM testing is only recommended by the WHO to assist diagnosing PLHIV with symptoms, those with CD4 cell count of less than 100 cells/mm3 in outpatient settings with the Alere Determine LAM assay.

Detection of *M.tb* LAM antigen in urine was performed using the 3rd generation SILVAMP TB LAM II (Fujifilm, Japan) assay following the manufacturing instructions for use (IFU). Briefly 200 µL of urine was transferred to the reagent tube, mixed and incubated at room temperature for 40 minutes. This was followed by the addition of 1 drop after mixing into the sample well of the cassette and pushing the “Push button 2”. The cassette was incubated until the “next” signal turned orange (15 minutes), and the “Push button 3” was activated within 30 minutes for a final incubation of 10 minutes before reading the results. Test results were interpreted by an operator who was blind to other diagnostic test results.

Computer aided detection (CAD) of chest x-rays (CXRs)

CAD algorithms interpret chest x-rays and provide a TB abnormality score ranging from 0.01 to 0.99 (or 1–100) as an output. This score is derived from the presence and degree of signs consistent with TB (cavitation, consolidation nodules, etc). The higher the score, the greater the radiological involvement and likelihood of the person having TB. The WHO recommends CAD for the interpretation of chest x-rays in individuals aged 15 years and older in the absence of a human reader. In Start4All phase 1, we will use the Qure.ai CAD software, zXR v4 for cloud uploads and v3.2 for offline uploads.

Pooled testing *of sputum with GeneXpert Ultra*

Pooled testing consists of combining equal volumes from multiple individuals’ samples and testing them together using a single test. If the pooled test result is negative, all individuals are presumed not to have the disease. If positive, each sample is retested individually to identify who has the disease.

Pooled testing was evaluated using the GeneXpert Ultra assay (Cepheid, USA). Sputum samples were first tested individually according to the manufacturer’s instructions. Briefly, each sample was mixed with sample reagent in a 2:1 ratio, vortexed or manually shaken, left to stand for 10 minutes, shaken again briefly, and left for a further 5 minutes before 2 mL was loaded into an Xpert cartridge for testing.

Equal amounts (typically 1–1.5 mL) from 2, 3, or 4 leftover processed sputum specimens were then combined in a new sterile sputum container to create pools. The pooled mixture was then gently mixed, and 2 mL was transferred into a new cartridge for testing. Pool size varied depending on the number of available specimens and access to refrigeration for

short-term storage. Pools were created using consecutive samples, and laboratory technicians performing the pooled testing were blinded to individual Ultra results.

Supplementary material 4: Full details of the Bayesian model for Start4All

Analysis by country:

Let:

- N be the number of participants in the study,
- $m = 5$ be the number of tests / assays being evaluated,
- $T_i, i = 1, \dots, N$ be a binary variable recording the TB status of the ith study participant (i.e. the result of the microbiological confirmation test),
- $X_{j,i}, j =$ cxr, crp, lam, pXpert, sXpert, $i = 1, \dots, N$ be the individual test results for the different participants,
- $p_{se,j}, p_{sp,j}$ be the sensitivity and specificity of test j,
- p_{TB} be the prevalence of TB in the population.

With this notation, we can write down the assumptions of the Bayesian MCMC estimation:

1. TB status is Bernoulli distributed.

$$T_i \sim \text{Bernoulli}(p_{TB})$$

with $\text{logit}(p_{TB}) = \beta_0 + \beta_s \cdot \text{sex} + \beta_h \cdot \text{hiv} + \beta_a \cdot s(\text{age})$, assuming weakly informative $\mathcal{N}(0{,}10)$ priors for the β_j parameters. $s(\,)$ indicates a restricted cubic / natural spline with 5 knots and β_a is the corresponding vector of parameters. For the analyses stratified by HIV status, the HIV term is dropped from the linear predictor term. We assume weakly informative normal $\mathcal{N}(0{,}10)$ priors for the coefficients of the linear predictor term. We assume that the sex and hiv parameters follow Bernoulli distributions, with weakly informative Kerman beta distribution priors (1) for their probability parameters.

2. Individual test results are Bernoulli distributed, conditional on TB status.

$$X_{j,i} \sim \begin{cases} \text{Bernoulli}(p_{se,j}) & \text{if } T_i = 1 \\ \text{Bernoulli}(1 - p_{sp,j}) & \text{if } T_i = 0 \end{cases}$$

3. Sensitivities, specificities are specified as the inverse logits of real scalars.

$$\begin{aligned} p_{se,j} &= \text{logit}^{-1}(z_{se,j}) \\ p_{sp,j} &= \text{logit}^{-1}(z_{sp,j}) \end{aligned}$$

4. These, or in other words, the logits of sensitivities, specificities, follow a non-trivial joint multivariate normal distribution.

$$\mathbb{Z} = (z_{se,w4ss}, z_{sp,w4ss}, z_{se,cxr}, z_{sp,cxr}, \dots, z_{se,sXpert}, z_{sp,sXpert}) \sim MV\mathcal{N}(\mu, \Sigma)$$

5. Priors for the means of the logits are weakly informative.

$$\begin{aligned} \mu_{se,j} &\sim \mathcal{N}(0{,}10) \\ \mu_{sp,j} &\sim \mathcal{N}(0{,}10) \end{aligned}$$

6. Prior for the covariance matrix follow a standard prior distribution for covariance matrices.

$$\Sigma \sim \text{invWishart}(\Lambda, \eta) \qquad \Lambda = I,\ \eta = 2 \cdot m + 1$$

7. Assuming that all dependencies between tests are captured through the above joint distribution of sensitivities and specificities and through conditioning on TB status, then all other, conditional, probability parameters can be derived from the sensitivity and specificity parameters. However, as explained earlier, the assumption of conditional independence is most likely unreasonable. For this reason, for each conditional parameter $\pi|\mathbf{T}$ (where $\mathbf{T}$ denote all test results upon which the probability parameter is conditional on), we will assume:
   - $\pi|\mathbf{T} \sim \beta(a = K + 1/3, b = M - K + 1/3)$
   - M, K are the random variables for the count of records for which the parameter is being evaluated (M) and the count of positive results (K) respectively and they are derived from the MCMC process (specifically $T_i, X_{i,j}$).

The above distributional assumptions completely specify the Bayesian model we will use for the per-country analyses.

<u>Aggregated, cross-country analysis:</u>

For facility-based screening, we will combine the data from all Start 4 All partner countries to estimate a single set of performance metrics at i) lowest level of care and ii) district-level care.

There is a need to account for country-level variation in the estimation procedure described above.

Let:

- N be the number of participants in the study,
- $m = 5$ be the number of tests / assays being evaluated,
- $T_{l,i}$ be a binary variable recording the TB status of the ith study participant from country l,
- $X_{l,j,i}$ be the individual test result for participant i from country l for assay j,
- $p_{se,l,j}, p_{sp,l,j}$ be the sensitivity and specificity of test j in country l,
- $p_{TB,l}$ be the prevalence of TB in the population in country l.

where the indices i, j, l iterate over:

- $l =$ Bangladesh, Brazil, Cameroon, Kenya, Malawi, Nigeria, Viet Nam,
- $j =$ cxr, crp, lam, pXpert, sXpert,
- $i = 1, \dots, N$

With this notation, we can write down the assumptions for the Bayesian MCMC estimation:

2. TB status is Bernoulli distributed.

$$T_{l,i} \sim \text{Bernoulli}(p_{TB,l})$$

with $\text{logit}(p_{TB}) = \beta_0 + \beta_s \cdot \text{sex} + \beta_h \cdot \text{hiv} + \beta_a \cdot s(\text{age})$, assuming weakly informative $\mathcal{N}(0,10)$ priors for the β_j parameters. $s(\quad)$ indicates a restricted cubic / natural spline with 5 knots and β_a is the corresponding vector of parameters. For the analyses stratified by HIV status, the HIV term is dropped from the linear predictor term.

3. Individual test results are Bernoulli distributed, conditional on TB status.

$$X_{l,j,i} \sim \begin{cases} \text{Bernoulli}(p_{se,l,j}) & \text{if } T_{l,i} = 1 \\ \text{Bernoulli}(1 - p_{sp,l,j}) & \text{if } T_{l,i} = 0 \end{cases}$$

4. Sensitivities, specificities are specified as the inverse logits of real scalars.

$$\begin{aligned} p_{se,l,j} &= \text{logit}^{-1}(z_{se,j} + \tau_{l,j}) \\ p_{sp,l,j} &= \text{logit}^{-1}(z_{sp,j} + \nu_{l,j}) \end{aligned}$$

where $\tau_{l,j}, \nu_{l,j}$ are country-level random effects.

5. These, or in other words, the logits of sensitivities, specificities, follow a non-trivial joint multivariate normal distribution.

$$\mathbb{Z} = (z_{se,w4ss}, z_{sp,w4ss}, z_{se,cxr}, z_{sp,cxr}, \dots, z_{se,sXpert}, z_{sp,sXpert}) \sim MV\mathcal{N}(\mu, \Sigma)$$

6. Priors for the means of the logits are weakly informative.

$$\begin{aligned} \mu_{se,j} &\sim \mathcal{N}(0,10) \\ \mu_{sp,j} &\sim \mathcal{N}(0,10) \end{aligned}$$

7. Prior for the covariance matrix follow a standard prior distribution for covariance matrices.

$$\Sigma \sim \text{invWishart}(\Lambda, \eta) \qquad \Lambda = I,\ \eta = 2 \cdot m + 1$$

8. Assuming that all dependencies between tests are captured through the above joint distribution of sensitivities and specificities and through conditioning on TB status, then all other, conditional, probability parameters can be derived from the sensitivity and specificity parameters. However, as explained earlier, the assumption of conditional independence is most likely unreasonable. For this reason, for each conditional parameter $\pi|\mathbf{T}$ (where $\mathbf{T}$ denote all test results upon which the probability parameter is conditional on), we will assume:
    - $\pi|\mathbf{T} \sim \beta(a = K + 1/3, b = M - K + 1/3)$
    - M, K are the random variables for the count of records for which the parameter is being evaluated (M) and the count of positive results (K) respectively and they are derived from the MCMC process (specifically $T_{l,i}, X_{l,j,i}$).
